# Supplementary material for: Transcriptomic Evidence of Mitochondrial Double‐Stranded RNA Accumulation in Brain Aging and Alzheimer's Disease
Source: Aging Cell. 2026 Jul 9;25(7):e70616. doi: 10.1111/acel.70616 (PMC13349986; doi:10.1111/acel.70616)
Supplement: Supplementary file 1 — Figure S1: (A–D) Additional analyses of the NABEC dataset (n = 105, see Table S1). (A) Combined average expression of genes related to mt‐RNA processing, metabolism or translation relative to younger subjects (< 35 years). Bars represent mean expression of genes (listed in A) relevant to each process ± SEM. **p adj < 0.005 compared to 36–45 year‐old group; Student's t‐test with Bonferroni correction. (B and C) Expression of nuclear encoded genes involved in (B) mt‐RNA processing, metabolism and translation, or (C) dsRNA sensing and related immune signaling relative to younger subjects (< 35 years). *p < 0.05, **p < 0.005 (Student's t‐test; unadjusted for multiple comparisons). (D) Relative expression of transcripts encoding mitochondrial pores and channels, and their regulators. *p adj < 0.05, **p adj < 0.005 (Student's t‐test with Bonferroni correction). Figure S2: (A) Predicted secondary structure of a single MT‐RNR2 transcript (via RNAfold) showing high‐probability intrastrand binding. (B) Ratio of antisense (L‐strand) to sense (H‐strand) reads for each age group to assess whether there is increased potential for interstrand binding. (C) Volcano plot of enriched (positive log2FC) and depleted (negative log2FC) PKR immunoprecipitated transcripts. (D–G) Quantification of A‐to‐I edits in mt‐RNA transcripts normalized to ChrM size factor in: (D) directly induced neurons (derived from fibroblasts) originating from younger (30–55 years) and older (> 56) humans; (E) human induced neuron, primary astrocyte and HMC3 microglia co‐cultures with and without mitochondrial fission (DRP1) inhibitor (P110); (F) vastus lateralis muscle from middle‐aged humans (40–64 years) before and after 4 months of daily treatment with 1000 mg Urolithin A; and (G) human embryonic lung fibroblasts subject to X‐ray induced senescence and/or knock‐out (−) of both BAK and BAX genes. Figure S3: (A–C) Functional analyses related to Figure 3. (A, B) Enriched Reactome, gene ontology biological process [file ACEL-25-e70616-s001.docx]

**SUPPLEMENTAL FIGURES AND TABLES**

**TITLE**

Transcriptomic evidence of mitochondrial double-stranded RNA accumulation in brain aging and Alzheimer’s disease

**AUTHORS**

Rachel L. Doser^1,2^ and *Thomas J. LaRocca^1,2^

**AFFILIATIONS**

1. Department of Health and Exercise Science, Colorado State University, Fort Collins, CO, USA.

2. Columbine Health Systems Center for Healthy Aging, Colorado State University, Fort Collins, CO, USA.

*:Lead Contact - tom.larocca@colostate.edu

**ABSTRACT**

Mitochondria and inflammation are tightly linked in aging and Alzheimer’s disease (AD), and recent evidence implicates mitochondrial double-stranded RNA (mt-dsRNA) as a potential trigger of inflammation. We examined mt-dsRNA accumulation and dsRNA signaling in brain aging and AD using complementary human brain tissue and *in vitro* transcriptomic datasets by quantifying mitochondrial transcripts, dsRNA editing, and related gene expression patterns. We found that mt-dsRNA signatures increased after midlife and coincided with reduced expression of mitochondrial RNA processing and translation machinery, along with increased expression of dsRNA antiviral signaling proteins, consistent with cytoplasmic mt-dsRNA-driven inflammation. In AD brains, mt-dsRNA accumulation was further increased and correlated with cognitive impairment, neuropathological severity, and AD risk genotypes. Genes associated with these measures reflected altered ubiquitin-dependent regulation of antiviral signaling, potentially indicating altered sensitivity to mt-dsRNA. Together, these findings highlight mitochondrial RNA homeostasis as an unrecognized contributor to age- and AD-related neurodegeneration and identify mt-dsRNA as a potential driver of chronic inflammation in the brain.

**SUPPLEMENTAL FIGURES**

**Supplemental Figure 1:** A-D) Additional analyses of the NABEC dataset (n=105, see Table S1). A) Combined average expression of genes related to mt-RNA processing, metabolism or translation relative to younger subjects (<35 yrs). Bars represent mean expression of genes (listed in A) relevant to each process ± SEM. **: p_adj_ <0.005 compared to 36-45 y.o. group; Student’s t-test with Bonferroni correction B and C) Expression of nuclear encoded genes involved in B) mt-RNA processing, metabolism and translation, or C) dsRNA sensing and related immune signaling relative to younger subjects (<35 years). *:p<0.05, **:p<0.005 (Student’s t-test; unadjusted for multiple comparisons). D) Relative expression of transcripts encoding mitochondrial pores and channels, and their regulators. *:p_adj_<0.05, **:p_adj_<0.005 (Student’s t-test with Bonferroni correction).

**Supplemental Figure 2:** A) Predicted secondary structure of a single MT-RNR2 transcript (via RNAfold) showing high-probability intrastrand binding. B) Ratio of antisense (L-strand) to sense (H-strand) reads for each age group to assess whether there is increased potential for interstrand binding. C) Volcano plot of enriched (positive log2FC) and depleted (negative log2FC) PKR immunoprecipitated transcripts. D-G) Quantification of A-to-I edits in mt-RNA transcripts normalized to ChrM size factor in: D) directly induced neurons (derived from fibroblasts) originating from younger (30-55 yrs) and older (>56) humans; E) human induced neuron, primary astrocyte and HMC3 microglia co-cultures with and without mitochondrial fission (DRP1) inhibitor (P110); F) vastus lateralis muscle from middle-aged humans (40-64 yrs.) before and after 4 months of daily treatment with 1000 mg Urolithin A; and G) human embryonic lung fibroblasts subject to X-ray induced senescence and/or knock-out (-) of both BAK and BAX genes.

**Supplemental Figure 3:** A-C) Functional analyses related to Figure 3. A and B) Enriched KEGG Reactome, gene ontology biological processes (GO:BP) and molecular function (GO:MF) terms for (A) the top 150 up- or down-regulated AD-DEGs (Figure 3B) and (B) WGCNA gene modules 1, 6 and 10 (Figure 3D). Genes from each module with a module membership greater than 0.9 were included in the gene ontology analysis. C) MitoCarta processes in which module 1 and 10 genes are enriched (using a hypergeometric overlap between gene lists).

**Supplemental Figure 4:** A) Volcano plot of differentially expressed genes in AD compared to NCI subjects (light orange data points, raw p-value < 0.01; dark orange data points indicate genes with FDR < 0.05). B) Enriched KEGG Reactome, GO:BP and GO:MF terms for genes differentially expressed (based on raw p-value < 0.01) in AD individuals when controlling for APOE genotype. C) Quantification of mt-dsRNA signatures grouped by low (e.g., e2/e2 or e2/e3), normal (e3/e3) or high (e3/e4 or e4/e4) genotype within NCI and AD subjects. #: p<0.05, *: p<0.002 (Bonferroni adjusted α).

**SUPPLEMENTAL TABLES**

**Table S1:**

| **NABEC Dataset Subject Characteristics** | | | | |
| --- | --- | --- | --- | --- |
| **Age Group** | **n =** | **Sex** | **Age** |  |
| <35 | 26 | 62% M / 38% F | 30.00 ± 3.84 |  |
| 36-45 | 36 | 72% M / 28% F | 41.19 ± 2.72 |  |
| 46-55 | 27 | 66% M / 33% F | 49.74 ± 2.67 |  |
| >56 | 16 | 81% M / 19% F | 66.81 ± 9.48 |  |

**Table S2:**

| **ROSMAP Dataset Subject Characteristics** | | | | | | | | |
| --- | --- | --- | --- | --- | --- | --- | --- | --- |
| **Group** | **n =** | **Sex** | **Age at death** | **Education (total yrs)** | **% with APOE e4** | **MMSE** | **Braak** | **Cerad** |
| NCI | 72 | 75% M 25 % F | 87.60 ± 4.15 | 16.06 ± 3.17 | 16.6% | 27.64 ± 2.53 | 3.19 ± 1.56 | 2.57 ± 1.23 |
| MCI | 38 | 71% M 29% F | 87.95 ± 3.34 | 15.10 ± 2.88 | 7.9% | 24.97 ± 3.64 *** | 3.52 ± 1.31 | 2.07 ± 0.99 |
| AD | 106 | 79% M 21% F | 88.87 ± 2.45 | 15.34 ± 3.09 | 29.2% | 12.06 ± 9.01 *** | 4.34 ± 0.98 *** | 1.66 ± 0.87 *** |

**Table S3:**

| **ROSMAP Metadata Subset Subject Characteristics** | | | | | | | | | | | | | |
| --- | --- | --- | --- | --- | --- | --- | --- | --- | --- | --- | --- | --- | --- |
| **Group** | **n =** | **Sex** | **Age at death** | **Educ. (total yrs)** | **MMSE** | **Braak** | **Cerad** | **Global**  **Cognition** | **Episodic Memory** | **Semantic**  **Memory** | **Working**  **Memory** | **Percep. Orientation** | **Speed Percep.** |
| NCI | 3 | 33% M 66 % F | All > 90 | 13.33 ± 1.15 | 28.00 ± 1.00 | 3.00 ± 0 | 3.00 ± 1.00 | 0.04 ± 0.16 | 0.28 ± 0.10 | 0.23 ±  0.52 | 0.04 ± 0.52 | -0.11 ±  0.31 | -0.46 ±  0.71 |
| MCI | 4 | 25% M 75% F | All > 90 | 11.5 ± 1.73 | 25.25 ± 3.20 | 3.50 ± 1.73 | 2.00 ± 0.81 | -0.73 ± 0.64 | -0.39 ± 0.37 | -0.35 ±  0.76 | -0.56 ± 0.24 | -0.67 ±  0.49 | -1.14 ±  1.14 |
| AD | 7 | 43% M 57% F | 89.83 ± 0.30 | 14.42 ± 1.98 | 10.43 ± 8.71* | 1.11 ± 0.09 | 2.00 ± 0.81 | -1.58 ± 0.61* | -1.71 ± 0.91* | -1.49 ± 0.88* | -1.02 ± 0.52* | -0.73 ±  0.94 | -1.91 ±  0.38* |
